# Evaluation of the Polyphenolic Composition and Bioactivities of Three Native Cabo Verde Medicinal Plants

**DOI:** 10.3390/ph15091162

**Published:** 2022-09-19

**Authors:** Anyse P. Essoh, Ângela Liberal, Ângela Fernandes, Maria Inês Dias, Carla Pereira, Filipa Mandim, Margarida Moldão-Martins, Pedro Cravo, Maria Paula Duarte, Mónica Moura, Maria M. Romeiras, Lillian Barros

**Affiliations:** 1 Linking Landscape, Environment, Agriculture and Food (LEAF), Associated Laboratory TERRA, Instituto Superior de Agronomia (ISA), Universidade de Lisboa, Tapada da Ajuda, 1340-017 Lisbon, Portugal; 2 Research Centre in Biodiversity and Genetic Resources (CIBIO), InBIO Associate Laboratory, Pole of Azores, Faculdade de Ciências e Tecnologia, Universidade dos Açores, 9500-321 Ponta Delgada, Portugal; 3 Universidade de Cabo Verde, Santiago, Praia CP 379, Cape Verde; 4 Centro de Investigação de Montanha (CIMO), Instituto Politécnico de Bragança, Campus de Santa Apolónia, 5300-253 Bragança, Portugal; 5 Laboratório Associado para a Sustentabilidade e Tecnologia em Regiões de Montanha (SusTEC), Instituto Politécnico de Bragança, Campus de Santa Apolónia, 5300-253 Bragança, Portugal; 6 Global Health and Tropical Medicine, Instituto de Higiene e Medicina Tropical, Universidade NOVA de Lisbona, 1349-008 Lisbon, Portugal; 7 Centro de Engenharia Mecânica e Sustentabilidade de Recursos (MEtRICs), Faculdade de Ciências e Tecnologia, Universidade NOVA de Lisboa, 2829-516 Caparica, Portugal; 8 Centre for Ecology, Evolution and Environmental Changes (cE3c) & CHANGE—Global Change and Sustainability Institute, Faculdade de Ciências, Universidade de Lisboa, 1749-016 Lisbon, Portugal

**Keywords:** bioactive properties, Cabo Verde, endemic medicinal plants, traditional medicine

## Abstract

The use of medicinal plants in a variety of health conditions remains essential for the discovery of new treatments. The present study aimed to investigate the bioactive properties of three native plants from Cabo Verde Islands, namely *Artemisia gorgonum* Webb, *Sideroxylon marginatum* (Decne. ex Webb) Cout., and *Tamarix senegalensis* DC., contributing to the characterization of less-known medicinal plants and their potential benefits for human health. Known compounds, such as kaempferol, quercetin, caffeyolquinic, and apigenin derivatives, among others, were detected in the plant species under study. Overall, all species demonstrated good antioxidant capacity, especially the ethanolic extracts of *A. gorgonum* (EC50 = 0.149 mg/mL) in TBARS assay. Moreover, the ethanolic extracts of the studied plants showed cytotoxic properties against tumor cells, and again the *A. gorgonum* extract proved to be the most effective in inhibiting tumor growth, mainly in the CaCO_2_ (GI50 = 17.3 μg/mL) and AGS (GI50 = 18.2 μg/mL) cell lines. Only the ethanolic extracts of *T. senegalensis* and *S. marginatum* demonstrated anti-inflammatory activity, albeit weak (EC50 = 35 and 43 μg/mL, respectively). The present study contributed to increased knowledge about the bioactive properties of these plants commonly used in traditional medicine, some of which was discussed for the first time, opening new perspectives for their use in a wider range of health conditions, especially in African countries, where access to modern health care is more limited.

## 1. Introduction

Over the centuries, medicinal plants have played a major role in the health systems of many cultures throughout the world [1]. Not surprisingly, after the standardization of conventional medicine and pharmacology, medicinal plants continue to be crucial in providing key bioactive compounds that are useful in various fields, such as the agri-food, pharmaceutical, and cosmetic industries, among others [2].

In Africa, the use of native plants in traditional medicine is intrinsically linked to the cultural heritage of each country, with this ancient knowledge being passed from generation to generation [3], where the use of medicinal plants (e.g., barks, seeds, fruits, and other parts) still stands as an important means to tackle a wide range of health conditions [4], underscoring the importance of traditional medicine for the survival of humankind throughout history. Today, this is particularly relevant for developing countries, where healthcare systems with scarce resources struggle to manage disease situations in populations lacking access to up-to-date treatment options [5]. In some cultures, traditional healers are considered as more reliable and accessible than other conventional physicians [6]. However, due to a generalized misconception that natural products are non-toxic, traditional herbal preparations, considered “natural”, are frequently perceived as being safer than other industrialized pharmacological products. However that assumption may not hold true in every case, since the concentration, type of extract, among other factors, may define the beneficial and/or cytotoxic potential of a given plant species. [7].

Considering the benefits and popularization of traditional medicine, many African countries have allowed and encouraged the implementation of a hybrid system of medical care integrating both approaches [8]. In the Cabo Verde archipelago (situated in the North Atlantic Ocean, near the western African coast, *circa* 600 km west of Senegal) traditional medicine is heavily dependent on plants, standing as a basic component of the health system, especially for rural communities, where pharmaceutical assistance is still limited [8]. However, in Cabo Verde, the gradual improvement of the national health system combined with the increase in health care structures increasingly closer to the population [9] and the disappearance of older generations of traditional medicine practitioners culminated in a decreasing use of this type of medicine [10]. Nonetheless, as in other countries, integrative medicinal practices are common and encouraged by decision makers [11].

Plants of the genus *Artemisia* L., generally represented by shrubs, constitute one of the genera of the Asteraceae family, consisting of ca. 475 species ranging from the temperate and subtropical Northern Hemisphere to South Africa and South America [12]. Numerous species from the genus *Artemisia* have been shown to display different biological activities, such as anthelminthic, antimalarial, antirheumatic, antibacterial, antihepatitis, anticancer, antiinflamatory, and menstrual-related disorders [13,14,15], being subject to substantial research in the last decade. Although *Artemisia* has been recognized as a rich source of bioactive compounds [16], *Artemisia gorgonum* Webb, endemic to Cabo Verde (Table 1), has been scarcely studied [17,18,19,20].

The genus *Tamarix* L. (Tamaricaceae family) includes ca. 73 species, some of which are used for medicinal purposes mostly in Asia and Africa [21,22]. Alike other native species of Cabo Verde, *Tamarix senegalensis* DC, one of the few native trees (native non-endemic) of this archipelago (Table 1), is still poorly studied with no data in the literature referring to its bioactive properties [23].

Likewise, *Sideroxylon marginatum* (Decne. ex Webb) Cout. (Sapotaceae family) is an endemic and endangered tree of Cabo Verde (Table 1) [24] of which little is known regarding its therapeutic potential. However, some studies related to other species of the same genus (with ca. 81 accepted species), showed encouraging results, with some beneficial phytochemicals being identified. For instance, for *Sideroxylon obtusifolium* (Roem. & Schult.) T.D.Penn., whose native range is from central to tropical America [25], several studies have reported its use for both medicine and as a source of food [26,27].

The use of plants as medicines dates back to the times of humankind. In fact, since ancient times, men have sought opportunities to improve living and health conditions in natural reserves towards improving survival [28]. The long-term experience accumulated throughout the long history of established practices can nowadays be used to enrich current scientific knowledge. Scientific research and development can thus provide additional evidence about safety and efficacy of plant-based medical solutions [29,30].

The plants under study were selected according to their recognition in traditional medicine, as well as their commercialization in the markets. Despite the recognized variety of the Cabo Verde flora, it remains largely unexplored, resulting in a poor knowledge about medicinal plants and their beneficial effects on human health and well-being. Under this perspective, the present study aimed to identify the phenolic profile of different extracts of *A. gorgonum, S. marginatum,* and *T. senegalensis*, towards investigating their bioactive properties, thus contributing to the documentation of lesser known medicinal plants that are available in countries that are heavily dependent on their use.

## 2. Results and Discussion

### 2.1. Phenolic Compounds

The tentative identification of the phenolic compounds in *T. senegalensis, A. gorgonum*, and *S. marginatum* samples, retention times (Rt), maximum absorbance (**λ**max), pseudomolecular ion ([M − H]^−^), and the main ion fragments (MS^n^) are presented in Table 2. The individual phenolic compounds present in these species were tentatively identified based on the data presented and, when possible, in parallel with existing standard compounds and/or published literature. In *T. senegalensis*, among the six compounds detected and tentatively identified, five were flavonoids and one was a phenolic acid. Regarding flavonoids, **peaks 2** and **3** were quercetin derivatives tentatively identified as methylquercetin-sulphate (tamarixetin sulphate) (λmax, 347 nm; [M − H]^−^ at *m/z* 395) and methylquercetin hexoside (tamarixetin-3-*O*-hexoside) (λmax, 351 nm; [M − H]^−^ at *m/z* 477), respectively. Additionally, three kaempferol derivatives were also identified in this species, namely methylkaempferol (kaempferide) (**peak 4**; λmax, 352 nm; [M − H]^−^ at *m/z* 299), kaempferol methyl ether sulphate (**peak 5**; λmax, 357 nm; [M − H]^−^ at *m/z* 379), and kaempferol-*O*-hexurunoside (**peak 6;** λmax, 346 nm; [M − H]^−^ at *m/z* 461). The standard compounds used to identify these flavonoids were previously described by Sekkien et al. [31]. A ferulic acid sulphate derivative (**peak 1**; λmax, 303 nm; [M − H]^−^ at *m/z* 273) was determined to be a phenolic acid by comparing its chromatographic profile with previously described compounds [31,32]. The phenolic profile of *T. senegalensis* had been previously investigated by other authors [1,2,3,4], who reported the presence of several kaempferol and quercetin derivatives, among other compounds, in different extracts of this species.

Concerning the *A. gorgonum* species, a total of twelve phenolic compounds were detected, of which ten were phenolic acids and two were flavonoids. **Peaks 7**, **8**, **9**, **10**, **14**, **15, 16**, **17** and **18** were tentatively identified as caffeoylquinic acid derivatives by comparing their chromatographic behavior with available literature [33,34,35]. In particular, for **peaks 9** and **17,** the standard compound previously used for its identification [35] showed the same retention time of **peaks 10** and **18,** respectively, identified as the trans isoform, and **peak 9** and **17,** (with the identical chromatographic behaviour as the aforementioned peak) as the cis isoform. Additionally, melilotoside (**peak 12**; λmax, 291 nm; [M − H]^−^ at *m/z* 325) was also identified in the *A. gorgonum* ethanolic extract and infusion preparation. Regarding flavonoids, two apigenin derivatives were detected and tentatively identified by comparison with available data [35], as apigenin-6-*C*-Glc-4”-*O*-Glc (isosaponarin) (**peak 11**; λmax, 334 nm; [M − H]^−^ at *m/z* 593) and apigenin-6-*C*-Ara-8-*C*-Glc (schaftoside) (**peak 13**; λmax, 330 nm; [M − H]^−^ at *m/z* 563). In relation to *S. marginatum* species, four phenolic compounds were discovered and tentatively identified by comparing their retention time and UV spectrum with available standards, such as quercetin-*O*-hexosyl-deoxyhexosyl-pentoside (**peak 19**; λmax, 354 nm; [M − H]^-^ at *m/z* 741), quercetin-*O*-hexosyl-pentoside (**peak 20**; λmax, 354 nm; [M − H]^−^ at *m/z* 595), quercetin-*O*-hexosyl-deoxyhexoside (**peak 21**; λmax, 354 nm; [M − H]^−^ at *m/z* 609), isorhamnetin derivative (**peak 22**; λmax, 350 nm; [M − H]^−^ at *m/z* 463), and quercetin-*O*-deoxyhexoside (**peak 23**; λmax, 347 nm; [M − H]^−^ at *m/z* 447).

The quantification information of the phenolic compounds detected in the ethanolic and infusion preparations is depicted in Table 3. Overall, the aqueous extract proved to be more efficient in extracting phenolic compounds. Among them, *T. senegalensis* presented the maximum concentration in phenolic compounds (36.35 mg/g extract), followed by *A. gorgonum* (100 mg/g extract) and *S. marginatum* (9.5 mg/g extract). In both the ethanolic and aqueous extracts of *T. senegalensis,* the ferulic acid sulphate derivative is the most abundant compound (1.25 and 10.7 mg/g extract, for the ethanolic and aqueous extracts, respectively). The phenolic profile of *A. gorgonum* showed interesting concentrations of caffeoylquinic acid derivatives, with 4,5-di-*O*-caffeoylquinic acid representing the main phenolic acid in both the ethanolic extract and the infusion preparation (0.53 and 24.3, mg/g extract, respectively), followed by melilotoside (1.58 and 17.8 mg/g extract, respectively), which, when compared with the former compound, is present at a higher concentration in the infusion preparation. Finally, the quercetin-*O*-hexosyl-deoxyhexosyl-pentoside was the major compound found in *S. marginatum*, with both extracts presenting the highest concentration (1.33 and 4.5 mg/g extract, respectively).

### 2.2. Antioxidant Activity

The antioxidant activity of the ethanolic extracts and infusion preparations of *T. senegalensis*, *A. gorgonum*, and *S. marginatum* samples was studied by determining their capacity to inhibit lipid peroxidation and oxidative hemolysis. Results showed that there were significant differences between the different extracts (Table 4). Overall, the ethanolic extracts present higher antioxidant activity as determined by the tiobarbituric acid reactive substances (TBARS) and oxidative haemolysis inhibition (OxHLIA) assays, when compared to the infusion preparations, except for the ethanolic extract of *T. senegalensis* in the OxHLIA test, where the infusion preparation shows higher anti-hemolytic activity (10.4 µg/mL). The *A. gorgonum* ethanolic extract presented the higher antioxidant activity, with EC_50_ values of 0.149 mg/mL and 15 µg/mL in TBARS and OxHLIA assays, respectively. The remaining extracts also proved to inhibit lipid peroxidation, presenting lower EC_50_ values indicative of a good antioxidant capacity of the plants under study, with a maximum of 0.87 mg/mL for the infusion preparation of *S. marginatum*. It is also relevant to highlight that only the ethanolic extract of *A. gorgonum* (15 µg/mL) and the infusion preparation of *T. senegalensis* (10.4 µg/mL) present EC_50_ values at lower concentrations than the positive control Trolox (21.8 µg/mL) in the OxHLIA assay. The *S. marginatum* infusion preparation presented the highest concentration (115 µg/mL) required to reach 50% inhibition of oxidative hemolysis.

The antioxidant activity of the studied species is poorly studied, with only a few studies available. Ortet et al. [17], found that the ethanolic extract of *T. senegalensis* plant leaves and flowers presented antioxidant potential and identified the bioactive compounds responsible for this and other bioactivities, namely lignans, furfurans, artemetin, and sesquiterpene lactones. Regarding *A. gorgonum*, Ortet et al. [18] investigated the antioxidant potential of its volatile oil using the DPPH and TBARS assays, showing that it exerted higher antioxidant activity than then the ethanolic extracts reported in our present work, with EC_50_ values of 0.48 and 0.06 mg/mL for both the DPPH and TBARS assays, respectively. Although both extracts demonstrated good antioxidant capabilities, the differences between both works may be due to the type of extraction performed, given that volatile essential oils are much more concentrated by nature, and, therefore, contain more bioactive compounds than the other extracts. To the best of our understanding, there are no studies regarding the antioxidant activity of *S. marginatum*. However, some studies have been made with other species of the same genus, such as *S. mascatense* [39] and *S. obtusifolium* [40], where compounds with proven bioactive properties from each plant were tested for their antioxidant potential. Here, we relate the antioxidant activity of this and *T. senegalensis* to the presence of quercetin and kaempferol derivatives, whose occurrence has been associated with this and other bioactivities [38].

### 2.3. Cytotoxic Activity

The extracts obtained from the different plants were also examined for their cytotoxic activity against tumor and non-tumor cell lines. Results were depicted as GI_50_ values, which represent the extract concentration that results in 50% of cell growth inhibition (Table 4). Although all extracts displayed GI_50s_ higher than that of the positive control (elipticin), both ethanolic extracts and infusion preparations were efficient in preventing the development of the studied tumor cell lines, with the exception of *T. senegalensis* against the MCF-7 tumor cell line. The *A. gorgonum* ethanolic extract presented the lowest GI_50_ for all of the tested cell lines, particularly against CaCO_2_ cells (17.3 µg/mL). Generally, the AGS cell line was the most vulnerable to both ethanolic extracts and infusion preparations of all plant species, in contrast to the MCF-7 cell line that was susceptible to higher extract concentrations, with GI_50_ values over 400 µg/mL in the case of the *T. senegalensis* infusion preparation of *A. gorgonum*. The cytotoxicity of *T. senegalensis*, *A. gorgonum*, and *S. marginatum* against a non-tumor cell line (PLP2) was also assessed by the sulforhodamine B assay that allows the evaluation of the impact of the extracts on cell proliferation. We found that, except for the ethanolic extract of *T. senegalensis* that presents a GI_50_ value of 178 µg/mL, no other extract was cytotoxic at the tested concentrations (Table 2). There are a few reports regarding the cytotoxic potential of *T. senegalensis* extracts against some human tumor cell lines. For example, a study by Hassan et al. [41] showed that its aqueous extract caused decreased cell proliferation in vitro and angiogenesis in human breast (MCF-7) and colon (HCT 116) cell lines. The same authors also reported lower toxicity for normal cells, compared with cancer cells, which is in line with our results. Martins et al. [19] assessed the cytotoxic activity of three main components of the chloroform extract of *A. gorgonum* aerial parts, specifically arborescin, artemetin, and sesamin, on neuroblastoma (SH-SY5Y), hepatocarcinoma (HepG2), and nontumoral bone marrow stromal (S17) cell lines and found that this species may be a useful basis for identifying antitumor molecules. Once again, at the best of our knowledge, this is the first report on the cytotoxic ability of *S. marginatum* against tumor and non-tumor cell lines, with the AGS cell line showing the lower GI_50_ value (75 µg/mL) for the infusion preparation, and no toxicity for both extracts against the PLP2 cell line.

### 2.4. Anti-Inflammatory Activity

EC_50_ values were calculated by evaluating the ability of the *T. senegalensis*, *A. gorgonum*, and *S. marginatum* ethanolic extracts and infusion preparations to prevent 50% of nitric oxide (NO) production in the mouse macrophage cell line RAW264.7 (Table 4). Overall, only the ethanolic extracts of *T. senegalensis* and *S. marginatum* presented anti-inflammatory potential, with EC_50_ values of 35 and 43 μg/mL, respectively. The remaining extracts did not seem to display anti-inflammatory activity at the higher concentration (400 μg/mL). Since all the extracts displayed good antioxidant activity, whose mechanisms are intrinsically linked to the inflammatory response and the lower anti-inflammatory activity observed in the majority of the extracts may be due to opposed properties between different compounds within the extract, although additional studies are required to test this theory.

Although the anti-inflammatory properties of *T. senegalensis* have not been yet studied, other plants belonging to *Tamarix* genus, namely *T. aphylla* [42] and *T. gallica,* seemed to reduce paw edema in rats [43]; specifically, *T. smyrnensis* [44] has been shown to inhibit NO release and transcription of the iNOS, TNF-α, IL-1β, and IL-6 genes. Similarly, there is no information in the literature considering the anti-inflammatory activity of *A. gorgonum* and *S. marginatum*. However, Wang et al. [45] were able to isolate a bioactive compound from *A. annua*, named artemisinin, that is widely known for its ability to kill malaria parasites. In that study, the authors explored the anti-inflammatory ability of artemisinin in TPA-induced skin inflammation in mice, verifying a significant inhibition of the expression of the NF-κB reporter gene, induced by TNF-α in a dose-dependent manner, as well as TNF-α-induced phosphorylation and the degradation of IκBα. They also found that pretreatment of cells with artemisinin prevented TNF-α-induced expression of NF-κB target genes, such as anti-apoptotic (c-IAP1, Bcl-2, and FLIP), proliferative (COX-2, cyclinD1), invasion (MMP-9), angiogenesis (VEGF), and key inflammatory cytokines (TNF-α, iNOS, and MCP1). The authors also isolated artemisinin from *A. gorgonum*, providing a parallel with *A. annua*. Once again, at the best of our knowledge, this is the unique report on the anti-inflammatory activity of *T. senegalensis*, *A. gorgonum*, and *S. marginatum* extracts.

Despite other studies having explored the bioactivity of plants from the same genus, our results are suboptimal, which may be related to the species itself, growing conditions, synergisms between different compounds in plants or other unidentified factors that may influence the chemical composition of plants. Further studies regarding the phenolic composition and its mechanisms of action may shed further light on the bioactive properties of the plants under study.

## 3. Materials and Methods

Samples of *T. senegalensis*, *A. gorgonum*, and *S. marginatum* were obtained during field surveys performed in 2020 on Santiago Island, the largest island of the Cabo Verde archipelago and a specimen of each species was deposited at the herbarium of the LISC/University of Lisbon. Prospections were performed in central exchange markets (i.e., Assomada Market, Praia Market, and São Domingos Market) to identify the most traded medicinal species. Samples were kept in dry conditions, at room temperature, and protected from light. To enhance the superficial contact and maximize compound content in the solvent medium, dried plant samples (leaves and stems) were ground in a lab-scale blender (Bimby TM31, Vorwerk, Wuppertal, Germany) and manually riddled with the Analysette 3 Spartan gear (Fritsch, Idar-Oberstein, Germany). In Table 1, information regarding the studied species (e.g., taxonomy, distribution, ecology, and conservation status) and medicinal uses in Cabo Verde is described.

### 3.1. Extract Preparation

Ethanolic extractions were performed according to a previously described procedure [46]. Briefly, 1 gram (g) of each plant sample was twice suspended in 30 mL of ethanol and stirred at 150 rpm for 1 h at room temperature. Following extraction, suspensions were filtered with Whatman No. 4 paper and the solvent removed by reduced pressure using a rotary evaporator (Büchi R-210, Flawil, Switzerland). The dried extracts were weighed and stored until further analysis.

For infusion preparations, boiled distilled water (100 mL, heating plate, VELP scientific) was added to each of the plant species (2 g), left to rest for 5 min, and then filtered through Whatman filter paper No 4. The resulting extracts were frozen and lyophilized to ensure accurate dosing and a long shelf-life.

### 3.2. UPLC Analysis of Phenolic Compounds

The phenolic composition was evaluated in the ethanolic and infusion preparations of the plant species and re-dissolved in ethanol/water (80:20, *v/v*) solution and water, correspondingly, to a final concentration of 10 mg/mL. The analysis was done using a Dionex Ultimate 3000 UPLC (Thermo Scientific, San Jose, CA, USA), supplied with a DAD detector (280 and 370 nm as the chosen wavelength) and combined to an electrospray ionization mass detector (LC-DAD-ESI/MSn). Identification of each phenolic compound was focused on the chromatographic data obtained (retention time, UV-Vis spectra, and mass), and compared to existing standard compounds, or earlier defined data in the literature, using the Xcalibur^®^ software (ThermoFinnigan, San Jose, CA, USA). The quantitative assessment of the discovered compounds was achieved using 7-level calibration curves built on the UV signal of available standard compounds. When precise standards were not available, the calibration curves of the most comparable standards were used. Working procedures were previously described in detail by the authors [46]. Results are expressed in mg/g extract.

### 3.3. Bioactive Properties

#### 3.3.1. Antioxidant Activity

Antioxidant activity was assessed in ethanolic extracts and infusion preparations through two cell-based assays: the thiobarbituric acid reactive substances (TBARS) and the oxidative hemolysis inhibition (OxHLIA).

For the TBARS assay, both extracts were re-dissolved in ethanol and water, respectively, and then dilluted from 2.5 mg/mL to 0.0390 mg/mL concentrations. Lipid peroxidation inhibition in porcine (*Sus scrofa)* brain cell homogenates was estimated by the decline in TBARS formation and, consequently, in the color strength of malondialdehyde–thiobarbituric acid (MDA–TBA), measuring their absorbance at 532 nm. The inhibition extent (%) was calculated using the formula: [(A − B)/A] × 100%, where A and B are related to the absorbance of the control and extract sample, respectively [47]. Results were expressed in EC_50_ values (mg/mL), representing sample concentration that produces 50% antioxidant activity. Trolox (Sigma-Aldrich, St. Louis, MO, USA) was used as positive control.

The antihaemolytic activity of the extracts was estimated through the OxHLIA assay using red blood cells (RBCs) isolated from healthy sheep, as previously described [47]. An erythrocyte solution (2.8%, *v/v*; 200 µL) was combined with 400 µL of either extract solution (0.0938–3 mg/mL PBS), PBS (control), or water (for complete hemolysis). After pre-incubation at 37 °C for 10 min with stirring, AAPH was included (200 µL, 160 mM in PBS, from Sigma-Aldrich) and the optical density determined at 690 nm every ~10 min in a ELX800 microplate reader (Bio-Tek Instruments, Winooski, VT, USA) until full hemolysis.

Results were expressed as EC_50_ values (μg/mL), at Δ*t* of 60 min, representing the extract concentration required to keep 50% of the erythrocyte population from the hemolytic activity for 60 min. Trolox was also employed as a positive control.

#### 3.3.2. Cytotoxic Activity

The cytotoxic and hepatotoxic activities of the extracts were assessed within a range of concentrations from 400 to 6.25 μg/mL, as previously described [47] using the sulforhodamine B (Sigma-Aldrich, St. Louis, MO, USA) colorimetric assay against three human tumor cell lines: (AGS—gastric adenocarcinoma; CaCO_2_—colorectal adenocarcinoma; MCF-7—breast carcinoma) (Leibniz-Institut DSMZ). A non-tumor cell line (PLP2—primary culture of pig liver culture) was also tested. Ellipticine was applied as a positive control. The results were expressed as GI_50_ values (μg/mL), which translate the extract concentration accountable for 50% inhibition of cell proliferation.

#### 3.3.3. Anti-Inflammatory Activity

The anti-inflammatory activity of the extracts was measured within a range of concentrations from 400 to 6.25 μg/mL based on nitric oxide (NO) production by a lipopolysaccharide (LPS)-stimulated murine macrophage cell line (RAW 264.7). NO production was quantified based on the nitrite concentration utilizing the Griess Reagent System kit, which contains sulphanilamide, N-1-naphthylethylenediamine dihydrochloride, and nitrite solutions, based on previously published protocols [48]. Dexamethasone (50 mM) was used as a positive control and samples without LPS served as the negative control.

The nitric oxide generated was defined by reading absorbances at 540 nm (ELX800 Biotek microplate reader, Bio-Tek Instruments, Inc., Winooski, VT, USA) and by contrast with the standard calibration curve. Results were expressed as EC_50_ values (µg/mL), representing the extract concentration that triggers 50% of NO production inhibition.

### 3.4. Statistical Analysis

Results for the bioactive properties of plant samples are presented as means ± SD (*n* = 3), in cases where analyses were done in triplicate. Statistical analysis was done using SPSS v. 23.0 software for Windows (IBM Corp., Armonk, NY, USA) and the one-way analysis of variance (ANOVA), while different means were evalated using the Tukey’s HSD test with α = 0.05 when statistically significant differences were identified. A Student’s *t*-test was used when only two samples were assessed.

## 4. Conclusions

The use of medicinal plants as sources of different medicines has played a fundamental role in the preservation of human health and well-being for millennia, with more than half of the industry drugs being derived from natural compounds worldwide. Before the development of modern medicine, traditional medicine prevailed as the only medical option available to millions of people, mainly in developing countries in rural and urban communities in Africa.

In the current study, we have investigated the bioactive potential of three selected medicinal plants endemic to Cabo Verde (*A. gorgonum*, *S. marginatum,* and *T. senegalensis*), namely for their antioxidant, cytotoxic, and anti-inflammatory activities, as well as their phenolic profile, revealing the presence of different phenolic compounds, namely kaempferol, quercetin, caffeyolquinic, and apigenin derivatives, among others. Overall, the *A. gorgonum* ethanolic extract displayed the higher antioxidant and cytotoxic potential against CaCO_2_ and AGS tumor cell lines. With the exception of the ethanolic extract of *T. senegalensis,* no other plant extract presented cytotoxicity against the non-tumor cell line PLP2. A slight anti-inflammatory activity was observed for the ethanolic extracts of *S. marginatum* and *T. senegalensis.*

This study contributes not only to the documentation of the species under investigation but also to a growing knowledge about their bioactive properties, some of which were described for the first time in this work. Further research must now be carried out in order to best characterize these medicinal plants, namely in terms of identifying the bioactive compounds responsible for these and other bioactivities, thus contributing to their wider use in different health conditions, mainly in developing countries where access to health care is more limited.

## Figures and Tables

**Table 1:** Characterization of the plant species under study and their use in traditional medicine in Cabo Verde.

Status	Plant (Species/Family)	Distribution	Common Name	Ecology and Conservation	Indication	Route of Administration
Native (non-endemic)						
	*T. senegalensis* (Tamaricaceae)	Arabian Peninsula, northwestern Africa, and Cabo Verde (in all the islands except in Fogo)	Tarrafe	Tree that grows in saline soil, sandy, and sea shore (Not Evaluated-IUCN)	Cold treatment	Infusion; herbal baths are also added to a tea together with a spoonful of grog (alcoholic)
Endemic						
	*A. gorgonum* (Asteraceae)	Cabo Verde: Santo Antão, Santiago, and Fogo islands	Losna	Perennial shrub that occurs in altitude semi-arid to sub-humid zones. It is threatened by continued habitat loss (Endangered-IUCN)	Intestinal parasites, fever, uterus, and cramps	Infusion; alcoholic
	*S. marginatum* (Sapotaceae)	Cabo Verde: all the islands, except in Sal and Maio	Marmulano	Tree growing in steep escarpments and inaccessible places. It is threatened by continued habitat loss (Endangered-IUCN)	Bone fractures and pain	Infusion; alcoholic; topical

**Table 2:** Retention time (Rt), wavelengths of maximum absorption in the visible region (λmax), mass spectral data, and identification of the phenolic compounds present in the ethanolic extracts and infusion preparations of the *T. senegalensis*, *A. gorgonum*, and *S. marginatum*.

Peak	Rt (min)	λmax (nm)	[M − H]^−^ (*m/z*)	MS^n^ (*m/z*)	Tentative Identification	References
				*Tamarix senegalensis*		
1	7.15	303	273	229 (9),193(100),178(11),153(5)	Ferulic acid sulphate derivative	[33,34]
2	14.3	347	395	315(33),300(100),217(5)	Methylquercetin-sulphate (tamarixetin sulphate)	[34]
3	15.42	351	477	315(12),301(100)	Methylquercetin hexoside (tamarixetin-3-*O*-hexoside)	[34]
4	15.79	352	299	285(100),271(14)	Methylkaempferol (kaempferide)	[34]
5	16.3	350	379	299(35),284(100)	Kaempferol methyl ether sulphate	[33,34]
6	16.71	346	461	285(100)	Kaempferol-*O*-hexurunoside	[34]
				*Artemisia gorgonum*		
7	5.11	325	353	191(100),179(45),161(5),135(5)	3-*O*-Caffeoylquinic acid	Standard compound
8	9.6	325	353	173(100),179(23),191(14),161(5),135(5)	4-*O*-Caffeoylquinic acid	[35]
9	10.91	325	353	191(100),179(8),161(7),135(4)	*cis* 5-*O*-Caffeoylquinic acid	[35]
10	11.01	326	353	191(100),179(12),173(5),135(5)	*trans* 5-*O*-Caffeoylquinic acid	[35]
11	13.11	334	593	431(34),341(56),311(100),313(12),283(17)	Apigenin-6-*C*-Glc-4”-*O*-Glc (isosaponarin)	[35]
12	13.27	291	325	163(100)	Melilotoside	[35]
13	14.02	330	563	503(25),473(98)443(100),413(5),383(35)353(32)325(5)297(5)	Apigenin-6-*C*-Ara-8-*C*-Glc (schaftoside)	[35]
14	16.26	322	515	MS^2^: 353(100); MS^3^: 191(23),179(54),173(100),135(19)	1,4-di-*O*-Caffeoylquinic acid	[35]
15	16.3	321	515	MS^2^: 353(100); MS^3^: 191(100),179(51),173(13),135(5)	3,5-di-*O*-Caffeoylquinic acid	[35]
16	17.14	326	515	MS^2^: 353(100); MS^3^: 191(34),179(42),173(100),135(11)	4,5-di-*O*-Caffeoylquinic acid	[35]
17	18.41	327	515	MS^2^: 353(100); MS^3^: 191(98),179(88),173(100),135(14)	*cis* 3,4-di-*O*-Caffeoylquinic acid	[35]
18	18.97	326	515	MS^2^: 353(100); MS^3^: 191(97),179(85),173(100),135(12)	*trans* 3,4-di-*O*-Caffeoylquinic acid	[35]
				*Sideroxylon marginatum*		
19	14	354	741	301(00)	Quercetin-*O*-hexosyl-deoxyhexosyl-pentoside	[36]
20	14.49	354	595	301(00)	Quercetin-*O*-hexosyl-pentoside	[37]
21	15.03	354	609	301(00)	Quercetin-*O*-hexosyl-deoxyhexoside	Characterization
22	15.21	350	463	315(00)	Isorhamnetin derivative	DAD/MS
23	16.87	347	447	301(00)	Quercetin-*O*-deoxyhexoside	[38]

**Table 3:** Quantification (mg/g of extract) of the phenolic compounds present in *T. senegalensis*, *A. gorgonum*, and *S. marginatum* ethanolic extracts and infusion preparations (mean ± SD, *n* = 3).

Peak	Quantification (mg/g Extract)		*t*-Student’s Test *p*-Value
	Ethanolic	Infusion	
	*Tamarix senegalensis*		
1	1.25 ± 0.05	10.7 ± 0.03	<0.001
2	0.63 ± 0.02	1.17 ± 0.02	<0.001
3	0.95 ± 0.04	1.61 ± 0.03	<0.001
4	0.78 ± 0.03	1.53 ± 0.01	<0.001
5	1.44 ± 0.08	1.27 ± 0.06	<0.001
6	1.59 ± 0.06	1.90 ± 0.08	<0.001
Total Phenolic Acids	1.25 ± 0.05	10.70 ± 0.03	<0.001
Total Flavonoids	5.4 ± 0.2	7.47 ± 0.03	<0.001
Total Phenolic Compounds	13.3 ± 0.5	36.35 ± 0.01	<0.001
	*Artemisia gorgonum*		
7	0.55 ± 0.02	2.23 ± 0.06	<0.001
8	0.48 ± 0.01	2.90 ± 0.03	<0.001
9	1.54 ± 0.09	9.5 ± 0.2	<0.001
10	nd	6.0 ± 0.1	-
11	0.013 ± 0.001	0.53 ± 0.05	<0.001
12	1.58 ± 0.02	17.8 ± 0.4	<0.001
13	0.41 ± 0.01	4.56 ± 0.09	<0.001
14	0.73 ± 0.03	12.66 ± 0.07	<0.001
15	0.50 ± 0.02	15.2 ± 0.4	<0.001
16	0.53 ± 0.02	24.3 ± 0.2	<0.001
17	0.37 ± 0.01	1.89 ± 0.07	<0.001
18	0.23 ± 0.01	2.07 ± 0.01	<0.001
Total Phenolic Acids	6.48 ± 0.01	95 ± 1	<0.001
Total Flavonoids	0.42 ± 0.01	5.1 ± 0.1	<0.001
Total Phenolic Compounds	6.90 ± 0.01	100 ± 1	<0.001
	*Sideroxylon marginatum*		
19	1.33 ± 0.02	4.5 ± 0.3	<0.001
20	0.64 ± 0.01	1.22 ± 0.04	<0.001
21	0.56 ± 0.01	1.36 ± 0.04	<0.001
22	0.67 ± 0.01	1.42 ± 0.06	<0.001
23	0.56 ± 001	0.93 ± 0.02	<0.001
Total Phenolic Compounds	3.76 ± 0.01	9.5 ± 0.3	<0.001

nd: not detected; calibration curves used: ferulic acid (y = 633126x − 185462, *R*^2^ = 0.999, LOD (Limit of Detection) = 0.20 μg/mL and LOQ (Limit of Quantification) = 1.01 μg/mL, peak 1); quercetin-3-O-glucoside (y = 34843x − 160173, *R*^2^ = 0.9999, LOD = 0.21 µg/mL; LOQ = 0.71 µg/mL, peaks 2–6 and 19–23); chlorogenic acid (y = 168823x − 161172; *R*^2^ = 0.9999, LOD = 0.20 µg/mL and LOQ = 0.68 µg/mL, peaks 7–10 and 14–18); apigenin-6-C-glucoside (y = 107025x + 61531, *R*^2^ = 0.998; LOD = 0.19 µg/mL; LOQ = 0.63 µg/mL, peaks 11 and 13), and *p*-coumaric acid (y = 301950x + 6966.7, *R*^2^ = 0.9999, LOD = 0.68 µg/mL; LOQ = 1.61 µg/mL, peak 12). Significant differences (*p* < 0.001) between extracts were assessed by a Student’s *t*-test.

**Table 4:** Antioxidant activity, cytotoxicity, and anti-inflammatory activities of the *T. senegalensis*, *A. gorgonum*, and *S. marginatum* ethanolic extracts and infusion preparations (mean ± SD, *n* = 3).

		*T. senegalensis*	*A. gorgonum*	*S. marginatum*
Antioxidant activity				
TBARS (EC_50_; mg/mL) ^a^	Ethanolic	0.161 ± 0.005 ^b^	0.149 ± 0.003 ^c^	0.27 ± 0.01 ^a^
	Infusion	0.83 ± 0.02 ^a^	0.42 ± 0.02 ^b^	0.87 ± 0.05 ^a^
OxHLIA (EC_50_; µg/mL) ^b^ Δ*t* = 60 min	Ethanolic	39 ± 1 ^b^	15 ± 1 ^c^	99 ± 2 ^a^
	Infusion	10.4 ± 0.5 ^c^	102 ± 4 ^b^	115 ± 4 ^a^
Cytotoxicity over tumor cell lines (GI_50_ μg/mL) ^c^				
AGS	Ethanolic	85 ± 2 ^b^	18.2 ± 0.4 ^c^	116 ± 12 ^a^
	Infusion	208 ± 20 ^a^	67 ± 6 ^b^	75 ± 1 ^b^
CaCO_2_	Ethanolic	125 ± 4 ^b^	17.3 ± 0.2 ^c^	251 ± 12 ^a^
	Infusion *	>400	181 ± 10	285 ± 2
MCF-7	Ethanolic	149 ± 4 ^b^	57 ± 6 ^c^	201 ± 1 ^a^
	Infusion *	>400	129 ± 10	209 ± 4
Cytotoxicity over non-tumor cell lines (GI_50_ µg/mL) ^c^				
PLP2	Ethanolic	178 ± 3	>400	>400
	Infusion	>400	>400	>400
Anti-inflammatory activity (EC_50_ μg/mL) ^d^				
RAW 264.7	Ethanolic *	35 ± 1	>400	43 ± 4
	Infusion	>400	>400	>400

^a^ EC_50_: extract concentration corresponding to 50% of antioxidant activity (TBARS) or ^b^ extract concentration required to keep 50% of the erythrocyte population intact for 60 min (OxHLIA assay); ^c^ GI_50_: extract concentration responsible for 50% inhibition of growth of human tumor (AGS, CaCO_2_ and MCF-7) or non-tumor cell line (PLP2); ^d^ EC_50:_ extract concentration responsible for achieving 50% of the inhibition of NO-production. Trolox EC_50_ values: 5.4 ± 0.3 μg/mL (TBARS), 21.8 ± 0.2 μg/mL (OxHLIA, Δ*t* 60 min); Ellipticine GI_50_ values: 1.23 ± 0.03 μg/mL (AGS), 1.21 ± 0.02 μg/mL (CaCO_2_), 1.02 ± 0.02 μg/mL (MCF-7) and 1.4 ± 0.1 μg/mL (PLP2); Dexamethasone EC_50_ values: 6.3 ± 0.4 μg/mL (RAW 264.7). Statistically significant differences (*p* < 0.05) between samples were assessed by a one-way ANOVA, using Tukey’s significant difference (HSD), and are indicated by different letters; * for CaCo and MCF-7 (infusion extracts) and RAW 264.7 (ethanolic extract), significant differences (*p* < 0.001) between the two samples were assessed by a Student’s *t*-test. AGS—gastric adenocarcinoma; CaCO_2_—colorectal adenocarcinoma; MCF-7—breast carcinoma; PLP2—primary culture of pig liver cells; RAW 264.7—murine macrophage cell line.

## Data Availability

Data is contained within the article.
